# Does improved oleic acid content due to marker-assisted introgression of *ahFAD2* mutant alleles in peanuts alter its mineral and vitamin composition?

**DOI:** 10.3389/fpls.2022.942617

**Published:** 2022-07-29

**Authors:** Jignesh H. Kamdar, Mital D. Jasani, Ajay B. Chandrashekar, Pasupulati Janila, Manish K. Pandey, John J. Georrge, Rajeev K. Varshney, Sandip K. Bera

**Affiliations:** ^1^ICAR-Directorate of Groundnut Research, Junagadh, India; ^2^Department of Microbiology, RK University, Rajkot, India; ^3^International Crop Research Institute for Semi-Arid Tropics, Hyderabad, India; ^4^Christ College, Rajkot, India; ^5^Department of Bioinformatics, University of North Bengal, Siliguri, India; ^6^State Agricultural Biotechnology Centre, Centre for Crop & Food Innovation, Food Futures Institute, Murdoch University, Murdoch, WA, Australia

**Keywords:** peanut, high oleic, MAS, MABC, yield, breeding, *Arachis hypogaea*

## Abstract

Peanuts (*Arachis hypogaea* L.) with high oleic acid content have extended shelf life and several health benefits. Oleic, linoleic, and palmitic acid contents in peanuts are regulated by *ahFAD2A* and *ahFAD2B* mutant alleles. In the present study, *ahFAD2A* and *ahFAD2B* mutant alleles from SunOleic 95R were introgressed into two popular peanut cultivars, GG-7 and TKG19A, followed by markers-assisted selection (MAS) and backcrossing (MABC). A total of 22 MAS and three MABC derived lines were developed with increased oleic acid (78–80%) compared to those of GG 7 (40%) and TKG 19A (50%). Peanut kernel mineral and vitamin composition remained unchanged, while potassium content was altered in high oleic ingression lines. Two introgression lines, HOMS Nos. 37 and 113 had over 10% higher pooled pod yield than respective best check varieties. More than 70% recurrent parent genome recovery was observed in HOMS-37 and HOMS-113 through recombination breeding. However, the absence of recombination in the vicinity of the target locus resulted in its precise introgression along with ample background genome recovery. Selected introgression lines could be released for commercial cultivation based on potential pod yield and oleic acid content.

## Introduction

Peanut (*Arachis hypogaea* L.) is an important crop, being cultivated in more than a hundred countries worldwide. China, India, Nigeria, and United States are the leading producers (FAOSTAT, 2021). Peanut seed is a source of good quality protein, minerals, vitamins, tocopherols, etc. (Wang et al., 2013) other than oil or fat. It contains ∼50% oil and ∼25% protein. In Asian countries, 50% of the peanut produce is utilized for oil extraction, while the rest is used for various food purposes. In European countries, 80% of produce is used in direct or processed foods, and the rest is used for oil extraction. Peanut oil is also used for processing ready-to-use therapeutic food (RTUF). Ready-to-use therapeutic food, rich in vitamins and minerals, is used to treat severe childhood malnutrition (Ali et al., 2013) and HIV-Positive patients (Brown et al., 2015) in many countries. Good quality peanut oil is an essential requirement of consumers and industries. The fatty acid composition of the seed determines the quality of peanut oil (Bera et al., 2019). Two unsaturated fatty acids (UFA), oleic acid (mono-UFA) and linoleic acid (poly-UFA), constitute up to 80% of the total peanut fat, having an equal share (Janila et al., 2016). Palmitic acid contributes ∼10% of total fat, and the remaining 10% is contributed by five saturated fatty acids, namely, stearic, arachidic, gadoleic, behenic, and lignoceric acids (Bera et al., 2018a). Thus, peanut oil quality is mainly determined by three major fatty acids, namely, oleic acid, linoleic acid, and palmitic acid. Peanut oil is preferred as a cooking medium because of its higher UFA, which reduces low-density lipoprotein (LDL) cholesterol, primarily responsible for cardiovascular diseases (CVD) (Mensink, 2016). Additionally, linoleic acid is thermodynamically unstable and forms CVD-causing trans-fatty acids on heating. Apart from reducing LDL level, peanut oil maintains the level of high-density lipoproteins (HDL) (Bera et al., 2019) and suppress tumorigenesis (Giulitti et al., 2021). Oleic acid acts as an anti-inflammatory agent by activating different pathways of immune-competent cells (Carrillo et al., 2012). Diet rich in oleic acid also decreases obesity, type 2 diabetes, and hypertension (Arsic et al., 2019). Besides, the auto oxidative stability of oleic acid is 10-fold higher than linoleic acid (Nawade et al., 2019). Thus, peanut seeds and their products with higher oleic acid to linoleic acid ratio (O/L) will have longer shelf life than normal peanuts. Hence, improved peanut genotypes with higher oleic acid and lower linoleic and palmitic acids are crucial for oil with greater health benefits and enhanced shelf life. The first induced mutant line, F435, with high oleic acid content (80%) and low linoleic acid content (2%), was reported from United States (Norden et al., 1987). Since then, the high oleic acid content peanut varieties SunOleic 95R and SunOleic 97R have been developed using conventional breeding techniques (Gorbet and Knauft, 1997, 2000). Studies showed that two alleles viz. *ahFAD2A* and *ahFAD2B* regulated the conversion of oleic acid to linoleic acid (Jung et al., 2000a,b) in peanuts. Later, allele-specific (Chu et al., 2007, 2009), cleaved amplified polymorphic sequences (CAPS) (Chen et al., 2010) and now KASP assay markers (by ICRISAT^1^) were developed to select plants with *ahFAD2* mutant alleles in early generations. Chu et al. (2011) first ever developed a high oleic acid content variety, “Tifguard High O/L” using marker-assisted backcrossing. Molecular breeding presents overwhelming prospects for the rapid development of high oleic cultivars with high specificity and precision (Pandey et al., 2012; Janila et al., 2016; Jasani et al., 2021; Kamdar et al., 2021). In peanuts, molecular markers associated with various traits have been reported (Bera et al., 2013, 2014a,b; Goswami et al., 2013; Kamdar et al., 2014). In India, the first marker-assisted high oleic peanut varieties, Girnar 4 and Girnar 5, were released for commercial cultivation in 2020. They contain more than 78% oleic acid with an oleic to linoleic acid ratio of 17. Furthermore, several advanced breeding lines of high oleic peanuts are under different stages of testing for release; this could replace entire native peanut cultivation with high oleic peanut in a very short period. In the process, peanuts may lose their native composition of vitamins and minerals. Groundnut is the richest source of energy component like oil (fat), protein and carbohydrates. Additionally, it contains vitamins, minerals and antioxidant. It is an abundant source of protein with capability to meet 46% recommend daily allowance. Essential vitamins such as Vitamin B, C, E, and K are important for normal body growth, boost the immune system and improves the metabolism. Furthermore, minerals such as iron, zinc, copper, calcium, and magnesium and manganese and selenium are important for the cardiac disease, enhance the immunity system and it also has important property of anti-aging.

Groundnut with all the above essential vitamins, minerals also represents a high-calorie diet with 884 calories per 100 g of oil. However, so far, no reports are available on the effect of high oleic content on the native composition of vitamins and minerals. In the present study, we deployed molecular breeding approaches to introgress high oleic content in two Indian peanut varieties, namely, GG-7 and TKG-19A. The study aimed to understand the effect of high oleic acid on the native composition of vitamins and minerals.

## Material and methods

### Plant material

Two peanut varieties, namely, GG-7 and TKG-19A, were selected for introgression of high oleic content and high oleic to linoleic ratio. The genotypes were selected based on low oleic acid content and habit groups. The oleic acid contents in GG-7 and TKG-19A were 45% and 49%, respectively. The GG-7 is a high-yielding and late leaf spot (LLS) resistant Spanish bunch peanut released by Junagadh Agricultural University, Junagadh, India. It is a selection of S-206 × FESR-8 1–1–9-B-B, with a productivity of 2,149 kg/ha. It has ∼50% oil content and is mainly cultivated in the rainy season (Rathnakumar et al., 2013). The TKG-19A is a high-yielding and aflatoxin-resistant Spanish bunch peanut released by Dr. Babasaheb Sawant Konkan Krishi Vidyapeeth, Dapoli, India. It is a derivative of TG-17 × TG-1, with ∼45% oil content and productivity of 2,260 kg/ha. Besides, it has bold and attractive kernels [Hand Picked and Selected (HPS) grade]. It is mainly cultivated during the post-rainy season (Rathnakumar et al., 2013). The SunOleic 95R was used as a donor parent for the high oleic trait. It was the first high oleic peanut runner variety developed by the Florida Agricultural Experimental Station, United States. It carries both *ahFAD2* homozygous mutant alleles with high oleic acid content (≥80%); however, the yield is poor (Gorbet and Knauft, 1997).

### Molecular markers

Allele specific-polymerase chain reaction (AS-PCR) markers developed by Chen et al. (2010) were used to confirm the presence of *ahFAD2* mutant alleles in F_1_ and BC_1_F_1_ plants. In contrast, cleaved amplified polymorphic sequences (CAPS) markers developed by Chu et al. (2007, 2009) were used to select plants with *ahFAD2* mutant alleles under the homozygous condition in F_2_ and BC_1_F_2_ populations. The details of both the markers are given in Supplementary Table 1.

### DNA extraction and marker genotyping

Individual plants grown in the field were tagged, and fresh leaves were collected. The DNAs of all the progenies (F_1_s, F_2_s, BC_1_F_1_s, and BC_1_F_2_s) were extracted using the protocol described by Mace et al. (2003). The DNA quality was checked using 0.8% agarose gel. Quantification was done using Nano drop (ND-1000, Nano Drop products, DE, United States), and working concentrations were adjusted to 50 ng μL^–1^ for genotyping. Genotyping of the target population with AS-PCR and CAPS markers was done following the protocols described by Bera et al. (2019).

### Hybridization and generation advancement

**FIGURE 1:**
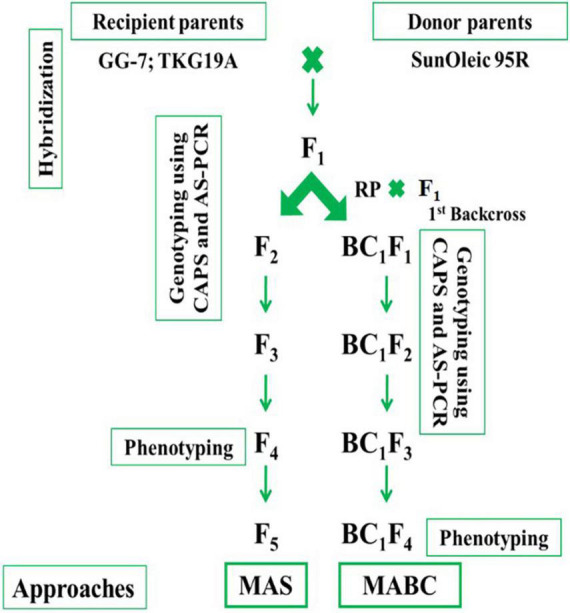
Schematic diagram of molecular breeding approaches.

The GG-7 and TKG-19A were used as female parents, while SunOleic 95R was used as donor parent. Hybridization between GG-7 × SunOleic 95R and TKG-19A × SunOleic 95R was accomplished in a glass house at ICAR-DGR, Junagadh, during the post-rainy season in 2016. Probably crossed seeds were planted in the 2017 rainy season. Later, the plants were genotyped with AS-PCR markers (Table 1) to identify F_1_ plants with *ahFAD2a* and *ahFAD2b* mutant alleles. A schematic diagram presenting the development and advancement of the progenies as well as the genotyping in different generations is provided in Figure 1.

**TABLE 1:** Pod yield of Spanish bunch (SB) high oleic breeding lines, percent increase of pod yield over check, shelling% and hundred kernels weight in the post-rainy season.

HOMS No.	Pooled pod yield/50 plants (g)	% pod yield increase over check	Shelling %	Hundred kernel weight (g)
116	476	–25	57	33
111	238	–63	49	32
80	336	–47	68	31
99	584	–8	60	47
3	600	–5	66	47
21	491	–23	62	52
74	393	–38	70	34
38	586	–8	60	47
37	784	24	60	47
6	445	–30	58	44
2	596	–6	68	58
101	538	–15	61	58
123	591	–7	54	30
109	361	–43	70	47
105	421	–34	72	36
103	374	–41	63	36
124	595	–6	57	34
GJG 32	634		68	39
SE	31.47	4.99	1.47	2.14
Interquartile Range	207		10.25	13.25

Disease scoring is not recorded as disease pressure is either less or nil during post rainy season.

Selected F_1_ plants with *ahFAD2* mutant alleles from both the crosses were selfed to obtain F_2_ progenies. Further, F_2_ progenies from both the crosses were planted in the 2017 post-rainy season and genotyped with AS-PCR and CAPS markers. The F_2_ plants with *ahFAD2* mutant alleles in homozygous status were identified and selfed. The selected single plant progenies were advanced to F_5_ generation using the single seed descent (SSD) method. Crosses, namely, GG-7 × SunOleic 95R and TKG-19A × SunOleic 95R were designated as MScross-I and MScross-II, respectively, for subsequent use.

In marker-assisted backcrossing, the F_1_ plants with *ahFAD2* mutant alleles from both the crosses (MScross-I and II) were used as the pollen donor. The female parents viz. GG-7 and TKG-19A were used as recurrent parents for the 1^st^ backcross (Figure 1). Probable crossed pods were harvested, and seeds were planted in the Net-house. Subsequently, they were genotyped with AS-PCR markers to identify BC_1_F_1_ plants with *ahFAD2a* and *ahFAD2b* mutant alleles. The BC_1_F_1_ plants carrying *ahFAD2* mutant alleles from both the crosses were selfed to obtain BC_1_F_2_ generation. Further, BC_1_F_2_ progenies of both the crosses were grown in the Net-house and genotyped with AS-PCR and CAPS markers. The BC_1_F_2–3_ plants with homozygous *ahFAD2* mutant alleles were identified, selfed, and advanced to BC_1_F_5_ generation following the single seed descent (SSD) method. Two backcrosses, namely, GG-7 × F_1_ (GG-7 × SunOleic 95R) and TKG-19A × F_1_ (TKG-19A × SunOleic 95R), were designated as MBCross-I and MBCross-II, respectively, for subsequent use.

### Analysis of oil content and fatty acid profile

The F_5_ seeds from MScross-I, MScross-II, MBCross-I, and MBCross-II were phenotyped for analyzing the fatty acid profile and oil content. Sound matured kernels from all the selected progenies were subjected to fatty acid and oil analysis using a gas chromatograph (model number GC-700, Thermo Fisher, United States) (Jokić et al., 2013) with 10 flame ionization detectors (FID). Oil content and fatty acid profile were estimated as per Bera et al. (2019).

### Yield and related traits of high oleic breeding lines

High oleic advanced breeding lines were tested for yield and related traits during the 2019 post-rainy and 2020 rainy seasons under field conditions. During the 2019 post-rainy season, 25 selected high oleic lines were tested separately for Spanish bunch (presence of flowers in main stem, erect, and generally short-lived) trial and Virginia bunch (absence of flowers in the main stem, semi-spreading, and generally long-lived) trial based on the growth habit.

Seventeen lines in the Spanish bunch trial and eight progenies in the Virginia bunch trial were planted along with respective high-yielding peanut varieties as control. For both the trials, each genotype was planted in a single line of a five-meter bed without replication due to less quantity of seed in each genotype. In the 2020 rainy season, both of these trials were repeated in RBD with two replications. Each genotype was planted in three lines of a four-meter bed. Observations on pod yield, shelling percent, and weight of hundred kernels were recorded during harvest. Besides, disease scoring for Late leaf spot and Rust diseases were noted from each progeny during both seasons. In the 2019 post-rainy season and 2020 rainy season, the pod yield of each genotype was calculated on 50 plants basis for further use and comparison. To compare the performances of high oleic progenies, recently released commercial varieties, GJG 32 and Girnar 4, were used as high yielding control for the Spanish bunch and Virginia bunch trials, respectively.

### Estimation of background genome recovery

Previously reported 347 polymorphic SSRs from 20 linkage groups were selected from the international consensus reference genetic map of peanut (Gautami et al., 2012) (Supplementary Table 4). The number of polymorphic markers per chromosome ranged from 8 (LG-b09) to 34 (LG-a01). These markers were deployed to determine recurrent parent genome (RPG) recovery in high yielding recombinant lines, i.e., HOMS-37 and 113. RPG recovery was analyzed using the formula: “RPG% = [{2(R) + (H)}/2N] × 100” (Bera et al., 2019); where “R” is the number of loci homozygous for recurrent parent allele; “H” is the number of loci remaining heterozygous, and “N” is the total number of polymorphic markers used in the background analysis.

### The nutrient profile of normal and high oleic peanut

Nutrient profiles of normal peanut varieties and their high oleic advanced breeding lines were compared to gauge the effect of enhanced oleic content on other native nutrients of the kernel. Major nutrient contents were analyzed for the seeds of the TKG 19A variety and two of its high oleic advanced breeding lines, namely, HFS-57 and HFS-59. Similar analyses were carried out for a highly farmers’ preferred peanut variety, GG 20, and its two high oleic advanced breeding lines, namely HFS-32 and HFS-38. These two high oleic breeding lines were previously developed in our laboratory. Sound mature kernels of six genotypes were used for nutrient profiling. Vitamins, namely, B1, B2, B3, B5, B6, B9, E, Choline, and Biotin, were analyzed, while the investigated elements were calcium, potassium, magnesium, zinc, iron, phosphorus, copper, and manganese. All the nutrients and vitamins were analyzed in three replications following standard methods and analysis was outsourced from CSIR – Central Food Technological Research Institute, Mysore, India, a premier Institute on Food Technology Research.

### Statistics

Univariate statistical parameters such as Standard error and 95% HPD regions were computed using PAST statistical software (Hammer et al., 2001). Introgression lines were compared with their parents for their mineral nutrient content using Student’s *t*-test using PAST statistical software (Hammer et al., 2001).

## Results

### Selection of peanut breeding lines with *ahFAD2* mutant alleles

#### Markers-assisted selection-derived lines

**FIGURE 2:**
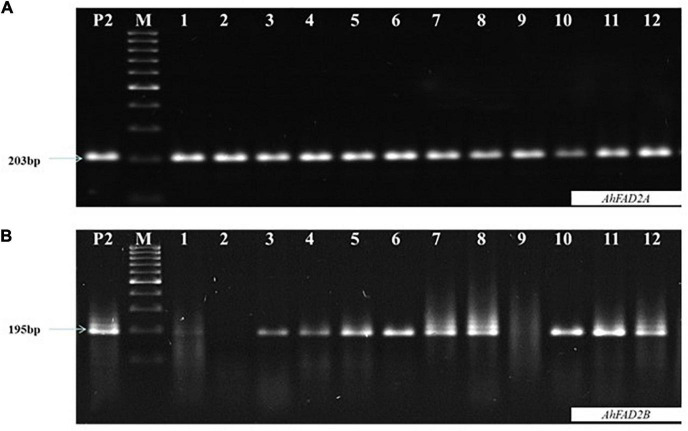
Representative image of allele specific-polymerase chain reaction (AS-PCR) **(A)** Showing amplification of *ahFAD2A* mutant allele (specific 203 bp amplification) **(B)** Showing amplification of *ahFAD2B* mutant allele (specific 195bp amplification); P2: SunOleic 95R, M:100bp DNA ladder.

**FIGURE 3:**
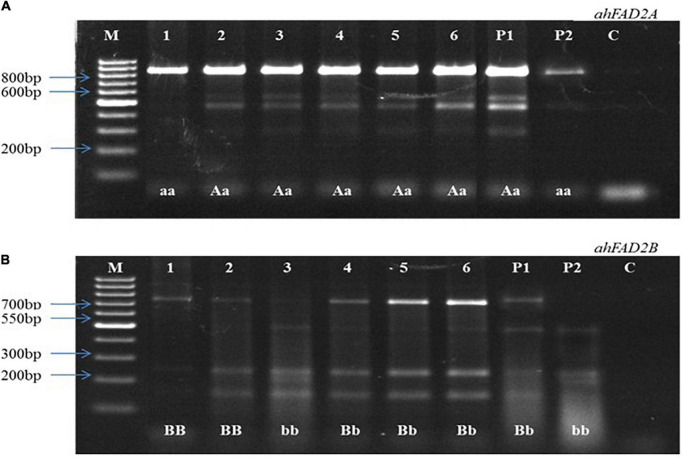
Representative image of cleaved amplified polymorphic sequences (CAPS). **(A)** Showing heterozygous and homozygous plants for *ahFAD2A* mutant allele, **(B)** Showing heterozygous and homozygous plants for *ahFAD2B* mutant allele. P1: female parent, P2: male parent, M: 100bp DNA ladder, C: Control, “AA, BB”: homozygous wild alleles, “Aa, Bb”: heterozygous alleles, and “aa, bb”: homozygous mutant alleles.

In the 2016 post-rainy season, hybridization was done for MSCross-I and MSCross-II, and 112 cross pods were harvested. F_1_ seeds were sown in the 2017 rainy season, and only 67 plants were available for genotyping while the rest did not germinate. These F_1_ plants were genotyped using AS-PCR markers. 23 F_1_ plants with *ahFAD2a* and *ahFAD2b* mutant alleles (i.e., 18 from MSCross-I and 5 from MSCross-II) were selected. These 23 F_1_ plants were selfed during the 2018 post-rainy season, and 381 F_2_ plants were obtained. All 381 F_2_ plants were genotyped with AS-PCR and CAPS markers to select F_2_ plants with homozygous *ahFAD2* mutant alleles (Figures 2, 3). Out of which 28 F_2_ plants (i.e., 23 from MSCross-I and 5 from MSCross-II) were found homozygous for both the mutant alleles. All F_2_ plants were advanced to F_5_ generation during the 2018 rainy, 2019 post-rainy, and 2019 rainy seasons. A total of 218 F_5_ single plant progenies (i.e., 184 from MSCross-I and 34 from MSCross-II) were developed. Fatty acid and oil content analyses were conducted in selected 126 F_5_ progenies out of 218 progenies (Supplementary Table 2). Rest 93 progenies originating from different F_2_ plants were not phenotyped for oil and fatty acid profile since most of the progenies from these particular F_2_ plants had normal oleic contents.

#### Markers-assisted backcrossing-derived lines

All 23 F_1_ plants identified with *ahFAD2* mutant alleles were used as pollen donors for the first backcross with the respective recurrent parents. From these crosses, 113 BC_1_F_1_ seeds, i.e., 53 seeds from MBCross-I and 60 seeds from MBCross-II, were harvested in the 2017 rainy season. All the BC_1_F_1_ seeds were sown in the 2018 post-rainy season and genotyped with AS-PCR markers. Five plants from MBCross-I and nine from MBCross-II were found with the *ahFAD2* mutant alleles. These plants were selfed, and altogether 63 BC_1_F_2_ seeds were harvested. All 63 BC_1_F_2_ seeds were planted during the 2018 rainy season and were genotyped with AS-PCR and CAPS markers. Six plants (two from MBCross-I and four from MBCross-II) were found homozygous for the *ahFAD2* mutant alleles. All BC_1_F_2_ plants were advanced to BC_1_F_4_ generation during the 2019 post-rainy and 2019 rainy seasons. Fatty acid and oil content analyses were done in all six F_4_ lines (Supplementary Table 2).

### Biochemical analyses of breeding lines with *ahFAD2* mutant alleles

#### Recombinant lines

GG-7 and TKG-19A contained 50% and 46% oil and 26% and 28% protein, respectively. Oil content ranged from 43 to 52%, while for protein content, the range was 25–29% for all the recombinant lines from both the crosses. The oleic acid ranged from 42 to 81% with an average of 68%, while the linoleic acid ranged from 3 to 34% with an average of 15%. We observed up to 6–102% increase in oleic acid content among the recombinant lines of GG-7 as compared to GG-7. The linoleic acid level was reduced by 6–92% in the recombinant lines of GG-7 compared to GG-7. The palmitic acid level declined by 1–53% in the recombinant lines of GG-7 compared to GG-7. Oleic to linoleic acid ratio increased up to 26 times in recombinant lines of GG-7 compared to GG-7.

On the other hand, there was an increase (8–61%) in oleic acid content among the recombinant lines of TKG-19A as compared to TKG-19A. The linoleic acid level was reduced (6–89%) in the recombinant lines of TKG-19A as compared to TKG-19A. Reduction (15–49%) was also observed for palmitic acid in the recombinant lines. The oleic to linoleic acid ratio increased up to 25 in the recombinant lines. In India, peanut genotypes having 78% or more oleic acid content are classified as high oleic peanut (AICRPG Proceeding 2019; Unpublished). In our study, 22 (eight from MScross-I and 14 from MScross-II) recombinant lines had ≥78% oleic acid content and oleic to linoleic acid ratio of ≥15 (Supplementary Table 2). Two recombinant lines, HOMS-111 and HOMS-37, were found promising in terms of quality traits. They had >50% oil content, >80% oleic acid content, ≥25 O/L ratios and 29% protein content.

#### Introgression lines

The ranges of oil and protein content among the six introgression lines produced under this study were 48–54% and 25–26%, respectively. Oleic acid ranged from 76 to 80%. Linoleic acid was found to be lower (4–5%.) in all the lines. O/L ratio ranged from 15 to 20, and palmitic acid content ranged between 6 and 8%.

There was a 95% increase in oleic acid content in the two introgression lines produced from GG-7, particularly with respect to the female parent. On the other hand, there was an 89% and 38% decrease in linoleic and palmitic acid, respectively, in these two introgression lines. O/L ratio, therefore, increased by 19–20 folds in these two introgression lines.

Oleic acid content increased by 52–60% in the four introgression lines produced from TKG-19A compared to the female parent. Linoleic and palmitic acid decreased by 85% and 50%, respectively, among the four introgression lines. O/L ratio increased up to15–20 folds as compared to the female parent.

Out of six introgression lines produced from the two crosses, HOMB-2, HOMB-4, and HOMB-6 had more than ≥78% oleic acid content (Supplementary Table 2). Among them, HOMB-2 had high oil (54%), high oleic acid (78%), and a high O/L ratio (20). Thus, a total of 25 high oleic (≥78% oleic acid content) breeding lines were developed using MAS and MABC strategies in the background of GG-7 and TKG-19. These 25 lines were also used in further studies.

### Yield performance of high oleic breeding lines

#### Post-rainy season of 2019

The SB trial check variety GJG-32 recorded 634 g of pod yield per 50 plants during the post-rainy season in 2019 (Table 1). None of the high oleic breeding lines performed better than GJG-32 except HOMS-37, which recorded >10% higher pod yield. Besides, HOMS-37 had 47 g hundred-kernel weight and 60% shelling out-turn.

The VB trial check variety Girnar 4 recorded 474 g of pod yield per 50 plants (Table 2). The high oleic VB breeding line, HOMS-10, had >10% higher pod yield than Girnar 4. Besides, HOMS-10 had 45 g of hundred-kernel weight and 56% shelling out-turn.

**TABLE 2:** Pod yield of Virginia bunch (VB) high oleic breeding lines, percent increase of pod yield over check, shelling% and hundred kernels weight in the post-rainy season.

HOMS No.	Pooled pod yield/50 plants (g)	% pod yield increase over check	Shelling %	Hundred kernel weight (g)
6	405	–15	61	35
114	371	–22	55	40
113	578	22	56	45
4	473	0	58	47
104	478	1	68	40
125	159	–66	50	32
122	368	–22	28	38
121	205	–57	32	26
Girnar-4	474	–	66	41
SE	44.98		4.67	2.16
Interquartile Range	189.5		22.5	9.5

Disease scoring is not recorded as disease pressure is either less or nil during post rainy season.

#### Rainy season of 2020

The SB trial check variety, GJG-32, recorded 96 g of pod yield per 50 plants during the rainy season in 2020 (Table 3). The seven high oleic breeding lines (HOMB-2, HOMS-21, HOMS-38, HOMS-101, HOMS-103, HOMS-116, and HOMS-123) had >10% higher pod yield than GJG-32. Out of seven breeding lines, HOMS-2 had the highest (74%) shelling out-turn, while HOMS-103 had the lowest (54%) shelling out-turn. Besides, HOMS-101 had the highest (42 g) hundred kernel weight while that for HOMS-116 (35 g) was the lowest.

**TABLE 3:** Pod yield of Spanish bunch (SB) high oleic breeding lines, percent increase of pod yield over check, shelling% and hundred kernels weight in the rainy season.

HOMS No.	Pooled pod yield/50 plants (g)	% pod yield increase over check	Shelling %	Hundred kernel weight (g)	LLS Score	Rust Score
116	122	28	57	35	5	3
111	43	–55	60	35	3	3
80	85	–11	59	37	3	1
99	87	–10	67	37	3	1
3	93	–3	64	35	3	2
21	111	16	59	40	3	1
74	68	–30	70	33	3	1
38	112	17	63	43	5	1
37	103	7	63	38	7	3
6	81	–16	66	41	3	1
2	108	13	74	37	5	2
101	135	41	66	42	7	2
123	129	34	67	40	5	2
109	100	5	71	39	3	3
105	80	–16	71	44	5	2
103	119	24	54	37	7	2
124	93	–3	55	38	7	2
GJG 32	96	–	61	35	2	0
SE	5.34		1.36	0.72	0.41	0.21
Interquartile Range	29.75		8.75	4.75	2.5	1.25

The VB trial check variety, Girnar 4, recorded 122 g pod yield per 50 plants (Table 4) in 2020, and the three high oleic breeding lines (HOMB-4, HOMS-113, and HOMS-125) had >10% higher pod yield than Girnar 4. Shelling percent (56–64%) and hundred kernel weight (32–45 g) were moderate among the three high oleic VB breeding lines.

**TABLE 4:** Pod yield of Virginia bunch (VB) high oleic breeding lines, percent increase of pod yield over check, shelling% and hundred kernels weight in the rainy season.

HOMS No.	Pooled pod yield/50 plants (g)	% pod yield increase over check	Shelling %	Hundred kernel weight (g)	LLS Score	Rust Score
6	101	–18	66	35	5	2
114	91	–25	57	36	5	2
113	147	21	64	45	3	1
4	161	32	64	37	3	2
104	133	9	68	44	5	2
125	139	14	56	32	2	1
122	106	–13	64	42	2	1
121	77	–37	56	26	2	0
Girnar-4	122	–	58	35	2	0
SE	9.27		1.56	2.2	0.46	0.28
Interquartile Range	47.0		80.5	9.5	30.0	10.5

#### Pooled performance over two seasons

The performance of the high oleic breeding lines was compared over the two seasons. The breeding lines: HOMS-37 (from the Spanish bunch habit group) and HOMS-113 (from the Virginia bunch habit group) had a >10% higher pod yield over respective checks (Tables 5, 6). HOMS-37 had a 21% higher pod yield than GJG-32, while pod yield in HOSM-113 was 22% higher than Girnar 4. Moreover, HOMS-37 contained 81% oleic acid, oleic to linoleic acid ratio of 25, 52% oil, and 29% protein content. In comparison, HOMS-113 contained 78% oleic acid, oleic to linoleic acid ratio of 20, 50% oil, and 28% protein content. Moreover, HOMS-37 had a 62% shelling out-turn and a hundred kernel weight of 42.5 g. Similarly, HOMS-113 had a 60% shelling out-turn and a hundred kernel weight of 45 g. Add LLS and rust reaction (Tables 5, 6). Besides, the recurrent parent genome recovery in selected high oleic breeding lines, HOMS-37 and HOMS-113, were tested using 374 SSRs anchored in 20 different linkage groups of cultivated peanut (Supplementary Table 4). Through recombination breeding, we observed more than 70% recurrent parent genome (RPG) recovery in HOMS-37 (75.1%) and HOMS-113 (72.8%) (Table 7). Further, we tested the recombination within the genomic region of target loci (*ahFAD2*) in both A and B genomes of high oleic breeding lines (HOMS 37 and HOMS 113) using 10 anchored SSRs (Table 8). Out of 10 SSRs, nine were expressed in both HOMS-37 and HOMS-113, indicating an absence of recombination close to the target location in these two high oleic breeding lines. This probably helps in increasing the oleic acid content above 78%.

**TABLE 5:** Pooled pod yield over two seasons of Spanish bunch (SB) high oleic breeding lines and percent increase of pod yield over check.

HOMS No.	Pooled pod yield/50 plants (g)	% pod yield increase over check	Shelling %	Hundred kernel weight (g)
116	299	–18	57	34
111	140	–62	55	34
80	210	–42	64	34
99	335	–8	64	42
3	346	–5	65	41
21	301	–17	61	46
74	231	–37	70	34
38	349	–4	62	45
37	443	21	62	43
6	263	–28	62	43
2	352	–4	71	48
101	336	–8	64	50
123	360	–1	61	35
109	231	–37	71	43
105	251	–31	72	40
103	246	–32	59	37
124	344	–6	56	36
GJG 32 (check)	365		65	37
SE	17.14		1.20	1.23
Interquartile Range	107.5		5.75	8.75

**TABLE 6:** Pooled pod yield over two seasons of Virginia bunch (VB) high oleic breeding lines and percent increase of pod yield over check.

HOMS No.	Pooled pod yield/50 plants (g)	% pod yield increase over check	Shelling %	Hundred kernel weight (g)
6	253	–15	64	35
114	231	–22	56	38
113	363	22	60	45
4	317	6	61	42
104	305	3	68	42
125	149	–50	53	32
122	237	–21	46	40
121	141	–53	44	26
Girnar-4 (check)	298		62	38
SE	25.00		2.71	1.94
Interquartile Range	121		13.5	8.5

**TABLE 7:** Details of homozygous/heterozygous SSRs found in recurrent parent genome recovery analysis.

Introgression line	No. of homozygous allele	No. of heterozygous allele	Total No. of polymorphic SSRs	RPG%
HOMS 37	158	188	346	72.83
HOMS 113	174	172	346	75.14

**TABLE 8:** Details of SSRs used for testing of recombination nearby target loci in selected two high oleic breeding lines.

Genome	Primer name	Linkage group	Position (cM)	HOMS 113	HOMS 37
A genome	gi-1107	a09	3.5	+	+
	PM-170	a09	13.9	+	+
	**ahFAD2a**	**a09**	**22.9**		
	AC3C07	a09	29	+	+
	TC5D06	a09	37.7	+	+
	Seq4G02	a09	40.7	+	+
B genome	Seq7G02	b09	62.6	+	+
	GM2120	b09	73.1	+	+
	Seq4G02	b09	78.8	+	+
	**ahFAD2B**	**b09**	**82.8**		
	GM1893	b09	90.1	+	+
	Seq17C09	b09	103.1	+	–

Indicates the targeted primer/allele.

### The nutrient profile of normal and high oleic peanut

TKG-19A and its high oleic advanced breeding lines, namely, HFS-57 and HFS-59, contained 50%, 79%, and 80% oleic acid content, respectively. In contrast, GG-20 and its high oleic content advanced breeding lines, namely, HFS-32 and HFS-38, contained 64%, 79%, and 80% oleic acid content, respectively. Mineral and vitamin profiles of normal oleic varieties and high oleic breeding lines were compared to understand the shift in nutrient profile due to the enhancement of oleic acid content in high oleic breeding lines (Tables 9, 10).

**TABLE 9:** Mineral compositions in the seeds of normal oleic groundnut varieties and their high oleic content breeding lines.

Genotype	Pedigree	Oleic acid content%	Calcium mg/100 g	Magnesium mg/100 g	Potassium mg/100 g	Zinc mg/100 g	Iron mg/100 g	Phosphorous mg/100 g	Copper mg/100 g	Manganese mg/100 g
TKG 19A	TG-17 × TG-1	49	130.0	109.42	732.22	20.63	10.74	478.49	00.32	10.14
HFS 57	TKG 19 A × SunOleic 95R	79	155.0	149.24	674.87	30.09	10.40	426.22	00.42	10.13
HFS 59	TKG 19 A × SunOleic 95R	80	148.0	111.13	674.90	20.91	10.63	410.30	00.26	10.02
		*	**NS**	**NS**	**	**NS**	**NS**	**NS**	**NS**	**NS**
GG 20	GAUG-10 × R-33-1	64	168.0	93.44	530.94	3.33	1.99	361.99	00.28	00.96
HFS 32	GG 20 × SunOleic 95R	79	198.0	111.71	580.18	20.71	20.29	408.50	10.33	10.12
HFS 38	GG 20 × SunOleic 95R	80	154.0	121.42	573.18	50.74	10.97	400.26	00.54	10.21
		*	**NS**	**NS**	*	**NS**	**NS**	**NS**	**NS**	**NS**

*, ** Significant difference @0.05, 0.01 probability level, respectively, between means of parents (TKG 19A, GG 20) and their progeny tested by t-test; NS, non-significant.

**TABLE 10:** Vitamin compositions in the seeds of normal oleic groundnut varieties and their high oleic content breeding lines.

Genotype	Pedigree	Oleic acid content%	Vitamin B1 mg/100 g	Vitamin B2 mg/100 g	Vitamin B3 mg/100 g	Vitamin B5 mg/100 g	Vitamin B6 mg/100 g	Vitamin B9 mg/100 g	Vitamin E mg/100 g	Choline mg/100 g	Biotin mg/100 g
TKG 19A	TG-17 × TG-1	49	10.52	BDL**of 0.1	11.30	10.30	BDL**of 0.1	00.22	50.64	22.5	00.01
HFS 57	TKG 19 A × Sunoleic 95R	79	10.23	BDL**of 0.1	90.57	10.05	BDL**of 0.1	00.25	70.10	17.5	00.01
HFS 59	TKG 19 A × Sunoleic 95R	80	20.01	BDL**of 0.1	11.40	00.98	BDL**of 0.1	00.28	70.91	16.3	00.01
		*	**NS**		**NS**	**NS**		**NS**	**NS**	**NS**	**NS**
GG 20	GAUG-10 × R-33-1	64	10.67	BDL**of 0.1	90.83	10.84	BDL**of 0.1	00.28	60.07	32.4	00.02
HFS 32	GG 20 × Sunoleic 95R	79	00.67	BDL**of 0.1	12.50	10.72	BDL**of 0.1	00.28	60.29	27.3	00.01
HFS 38	GG 20 × Sunoleic 95R	80	10.11	BDL**of 0.1	10.40	10.65	BDL**of 0.1	00.22	50.67	24.3	00.01
		*	**NS**		**NS**	**NS**		**NS**	**NS**	**NS**	**NS**

* Significant differences @0.05 probability level, respectively, between means of parents (TKG 19A, GG 20) and their progeny tested by t-test; NS, non-significant.

BDL**- Beyond Detection Level.

#### Calcium

Calcium content was 130 and 168 mg per 100 g of the kernel in TKG-19A and GG-20, respectively. It increased from 130 mg in TKG-19A to 155 mg in HFS-57 and 148 mg in HFS-59. Similarly, it increased from 168 mg in GG-20 to 198 mg in HFS-32 but decreased to 154 mg in HFS-38.

#### Potassium

Potassium content was 732.22 mg and 530.94 mg per 100 g of the kernel in TKG-19A and GG-20, respectively. Potassium content decreased from 737.22 mg in TKG-19A to 674.87 mg in HFS-57 and 674.9 mg in HFS-59. It increased from 530.94 mg in GG-20 to 580.18 mg in HFS-32 and 573.18 mg in HFS-38.

#### Magnesium

The magnesium content in TKG-19A and GG-20 was 109.42 mg and 93.44 mg per 100 g of the kernel, respectively. Magnesium content increased from 109.42 mg in TKG-19A to 149.24 mg in HFS-57 and 111.13 mg in HFS-59. Similarly, it increased from 93.44 mg in GG-20 to 111.71 mg in HFS-32 and 121.42 mg in HFS-38.

#### Phosphorous

The phosphorous content in TKG-19A and GG-20 was 478.49 mg and 361.99 mg per 100 g of the kernel, respectively. Phosphorous content decreased from 478.49 mg in TKG-19Ato 426.22 mg in HFS-57 and 410.30 mg in HFS-59. In contrast, it increased from 361.99 mg in GG-20 to 408.50 mg in HFS-32 and 400.26 mg in HFS-38.

#### Zinc

Zinc content in TKG-19A and GG-20 was 2.63 mg and 3.33 mg per 100 g of the kernel, respectively. Zinc content increased from 2.63 mg in TKG-19A to 3.09 mg in HFS-57 and 2.91 mg in HFS-59. Conversely, it decreased from 3.33 mg in GG-20 to 2.71 mg in HFS-32 and increased to 5.74 mg in HFS-38.

#### Iron

Iron content in TKG-19A and GG-20 was 1.74 mg and 1.99 mg per 100 g of the kernel, respectively. Iron content decreased from 1.74 mg in TKG-19A to 1.40 mg in HFS-57 and 1.63 mg in HFS-59. On the other hand, it increased from 1.99 mg in GG-20 to 2.99 mg in HFS-32 and 1.97 mg in HFS-38.

#### Copper

The copper content in TKG-19A and GG 20 was, respectively, 0.32 mg and 0.28 mg per 100 g of the kernel. Copper content increased from 0.32 mg in TKG-19A to 0.42 mg in HFS-57 while decreasing to 0.26 mg in HFS-59. Similarly, it increased from 0.28 mg in GG-20 to 1.33 mg in HFS-32 and 0.54 mg in HFS-38.

#### Manganese

The manganese content in TKG-19A and GG-20 was 1.14 mg and 0.96 mg per 100 g of the kernel, respectively. Manganese content was 1.14, 1.13, and 1.02 mg in TKG-19A, HFS-57, and HFS-59, respectively. Manganese content increased from 0.96 mg in GG 20 to 1.12 mg in HFS-32 and 1.21 mg in HFS-38.

#### Vitamin B1

Vitamin B1 content in TKG-19A and GG-20 was 1.52 mg and 1.67 mg per 100 g of the kernel, respectively. Vitamin B1 content decreased from 1.52 mg in TKG-19A to 1.23 mg in HFS-57, while it increased to 2.01 mg in HFS-59. Similarly, it decreased from 1.67 mg in GG 20 to 0.67 mg in HFS-32 and 1.11 mg in HFS-38.

#### Vitamin B2

Vitamin B2 in all the samples was below the detectable limit of 0.1 mg/100 g of a peanut kernel.

#### Vitamin B3

Vitamin B3 content in TKG-19A and GG-20 was 11.3 mg and 9.83 mg per 100 g of the kernel, respectively. Vitamin B3 content decreased in HFS-57 than TKG-19A, while it was stable in HFS-59. In contrast, it increased from 9.83 mg in GG-20 to 12.5 mg in HFS-32 and 10.4 mg in HFS-38.

#### Vitamin B5

Vitamin B5 content in TKG-19A and GG-20 was 1.3 mg and 1.84 mg per 100 g of the kernel. Vitamin B5 content decreased from 1.3 mg in TKG-19A to 1.05 mg in HFS-57 and 0.98 mg in HFS-59. Similarly, it decreased from 1.84 mg in GG-20 to 1.72 mg in HFS-32 and 1.65 mg in HFS-38, respectively.

#### Vitamin B6

Vitamin B6 in all the samples was below the detectable limit of 0.1 mg/100 g of a peanut kernel.

#### Vitamin B9

Vitamin B9 content in TKG-19A and GG-20 was 0.22 mg and 0.28 mg per 100 g of the kernel, respectively. Vitamin B9 content increased from 0.22 mg in TKG-19A to 0.25 mg in HFS-57 and 0.28 mg in HFS-59. On the other hand, it was similar in both GG-20 and HFS-32 while it decreased from 0.28 mg in GG-20 to 0.22 mg in HFS-38.

#### Vitamin E

Vitamin E content in TKG-19A and GG-20 was 5.64 mg and 6.07 mg per 100 g of the kernel, respectively. Vitamin E content increased from 5.64 mg in TKG-19A to 7.1 mg in HFS-57 and 7.91 mg in HFS-59. Similarly, it increased from 6.07 mg in GG-20 to 6.29 mg in HFS-32, while it decreased to 5.67 mg in HFS-38.

#### Choline

Choline contents in TKG-19A and GG-20 were, respectively, 22.5 mg and 32.4 mg per 100 g of the kernel. Choline content decreased from 22.5 mg in TKG-19A to 17.5 mg in HFS-57 and 16.3 mg in HFS-59. Similarly, it also decreased from 32.4 mg in GG-20 to 27.3 mg in HFS-32 and 24.3 mg in HFS-38.

#### Biotin

Biotin content in TKG-19A, GG-20, and their high oleic breeding lines were uniform and ranged from 0.01 to 0.02.

## Discussion

The main goal of the worldwide peanut breeding program is the development of high oleic acid content peanuts. The high oleic peanuts are preferred by food processing industries for improved shelf-life, as well as by consumers due to their multiple health benefits. The enzyme fatty acid desaturase-2 controls the conversion of oleic acid to linoleic acid. Homeologous genes *ahFAD2A* and *ahFAD2B* encode the enzyme fatty acid desaturase-2 in peanuts (Jung et al., 2000a,b; Yu et al., 2008; Kamdar et al., 2021). In peanuts, the oleic acid content gets increased if *ahFAD2A* and *ahFAD2B* genes are inactive due to mutations since they do not produce the enzyme fatty acid desaturase-2. The development of linked AS-PCR (Chen et al., 2010) and CAPS (Chu et al., 2007, 2009) markers for both the *ahFAD2* genes accelerated the high oleic peanut breeding programs. Earlier, nematode resistance (Simpson et al., 2003a,b), rust resistance (Varshney et al., 2014), and high oleic acid (Janila et al., 2016; Bera et al., 2018a,^2019^) traits were successfully transferred to peanut using molecular breeding. Both the markers were successfully used to track the *ahFAD2A*, and *ahFAD2B* mutant alleles in MAS and MABC approaches (Janila et al., 2016; Bera et al., 2018a,^2019^). These approaches significantly reduced the time and the required amount of breeding material in different generations. In the present study, two popular peanut varieties, namely, GG-7 and TKG-19A, were improved for high oleic acid content using molecular breeding techniques. The high oleic traits are introgressed from a mutant line SunOleic95R from the United States. SunOleic95R is reported as poor yielding in the United States and the same was also observed in Indian conditions (data not reported).

Genotyping was done in early generation (F_1_, F_2_, BC_1_F_1_, and BC_1_F_2_), and a large number of plants, not confirming the presence of *ahFAD2* mutant alleles, were rejected. Consequently, phenotyping was carried out in advanced generations (F_5_ and BC_1_F_4_) to select the promising high oleic acid content lines for further evaluation. We observed a higher number of high oleic lines in MAS (37 plants) than that in MABC (4 plants) among the progenies confirmed as homozygotes for both the *ahFAD2* mutant alleles. Similarly, Janila et al. (2016) observed a higher number of lines with high oleic acid content in MAS cross than that for MABC. Among the selected lines, 80 lines had more than 70% oleic acid content, which is expected to meet the need of the food processing industries. To produce high-quality oil, high oil with high oleic lines is ideal for the oil industry. It enhances the shelf-life and health benefits of peanut products. On the other hand, low oil content peanuts are used for table and confectionary purposes. In our study, seven lines were identified with high oil and high oleic acid content. Oil content in these lines ranged between 50 and 54%. Two lines were identified with low oil and high oleic acid content. Oil content in these lines ranged between 44 and 45%. The oleic acid content in Sunoleic 95R was 78.0%, and among the lines, it ranged from 59.40 to 80.69%. A similar variation in oleic acid content was also reported by Janila et al. (2016) and Bera et al. (2018a; 2018b). Variations in the oleic acid content probably indicate the presence of several gene modifiers determining the oleic acid content in peanuts. Modifier genes usually increase or decrease the expression of a major gene (Briggs and Knowles, 1967). Such genes are very difficult to characterize due to their small effect on the trait of interest (Hamdan et al., 2009). Earlier, variations in the oleic acid content due to modifier genes were reported in other oilseeds crops (Fernández-Martínez et al., 2004; Hamdan et al., 2012). We report that genotyping in early generations combined with phenotyping in advanced generations was found effective in selecting the lines with high oleic acid content. Our study found a lower level of linoleic acid in the lines with higher oleic acid content due to the inactivation of the ahFAD2 gene, which is responsible for oleic acid to linoleic acid conversion. In the recent past, Wang et al. (2015) reported that the level of palmitic acid was also affected by *ahFAD2A* and *ahFAD2B* mutant alleles. We also observed that the lines containing both the mutant alleles had a lower level of palmitic acid content. High oleic breeding, though, mostly focused solely on the oleic acid content in the improved lines. In this study, we successfully developed 41 lines in the background of GG-7 and TKG-19A with high oleic acid content and varying percentages of oil content (44–54%) (Supplementary Table 2). Peanut is rich source of different vitamins and minerals. Moreover, some of the highest quantities such as, Folate has a 60% RDA. Folate is an important vitamin required during pregnancy meanwhile it helps in the production and support of cells (Whitney and Rolfes, 2018). Niacin has a 75% RDA, niacin reduces the risk of heart diseases, Thiamine has a 53% RDA, Thiamine works as a cofactor for several metabolic enzymes. Manganese has an 84% RDA, which is also a cofactor for metabolic enzymes, copper has a 127% RDA, copper is producer of key proteins such as hemoglobin and collagen. Phosphorous has a 54% RDA, phosphorous is important for metabolism which maintains the strong bones. While considering recommended daily values of Vitamins such as, Folate, vitamin E, riboflavin, thiamine, biotin, niacin as well as minerals such as, iron, zinc, calcium, magnesium, copper and phosphorous illuminate the beneficial role of groundnut in a well-balanced diet. The study also reports minimum or no adverse effect of high oleic traits on quantity of native vitamins and mineral in introgression lines. A GWAS analysis conducted in 120 genotypes from the United States minicore collection led to identification of 24 QTLs for boron (B), 2 QTLs for copper (Cu), 6 QTLs for sodium (Na), 3 QTLs for sulfur (S), and 1 QTL for zinc (Zn) with 18.35–27.56% PVE. In addition, mining of genomic regions further discovered 110 casual candidate genes. Interestingly, *arahy.KQD4NT* (position 5,413,913–5,417,353) has been detected as the important elemental/metal transporter gene identified on chromosome B04 (Zhang et al., 2019). These developed introgression lines could either be released as varieties after further evaluation in national trials or used as donors in the new peanut breeding programs.

## Data availability statement

The original contributions presented in the study are included in the article/Supplementary material, further inquiries can be directed to the corresponding author.

## Author contributions

All authors listed have made a substantial, direct, and intellectual contribution to the work, and approved it for publication.

## Footnotes

1
https://excellenceinbreeding.org/module3/kasp
